# The Differences Between Individuals Engaging in Nonsuicidal Self-Injury and Suicide Attempt Are Complex (*vs.* Complicated or Simple)

**DOI:** 10.3389/fpsyt.2020.00239

**Published:** 2020-04-07

**Authors:** Xieyining Huang, Jessica D. Ribeiro, Joseph C. Franklin

**Affiliations:** Department of Psychology, Florida State University, Tallahassee, FL, United States

**Keywords:** nonsuicidal self-injury, suicide attempt, complexity, machine learning, differences

## Abstract

**Background:**

Why do some people engage in nonsuicidal self-injury (NSSI) while others attempt suicide? One way to advance knowledge about this question is to shed light on the differences between people who engage in NSSI and people who attempt suicide. These groups could differ in three broad ways. First, these two groups may differ in a simple way, such that one or a small set of factors is both necessary and sufficient to accurately distinguish the two groups. Second, they might differ in a complicated way, meaning that a specific set of a large number of factors is both necessary and sufficient to accurately classify them. Third, they might differ in a complex way, with no necessary factor combinations and potentially no sufficient factor combinations. In this scenario, at the group level, complicated algorithms would either be insufficient (*i.e.*, no complicated algorithm produces good accuracy) or unnecessary (*i.e.*, many complicated algorithms produce good accuracy) to distinguish between groups. This study directly tested these three possibilities in a sample of people with a history of NSSI and/or suicide attempt.

**Method:**

A total of 954 participants who have either engaged in NSSI and/or suicide attempt in their lifetime were recruited from online forums. Participants completed a series of measures on factors commonly associated with NSSI and suicide attempt. To test for simple differences, univariate logistic regressions were conducted. One theoretically informed multiple logistic regression model with suicidal desire, capability for suicide, and their interaction term was considered as well. To examine complicated and complex differences, multiple logistic regression and machine learning analyses were conducted.

**Results:**

No simple algorithm (*i.e.*, single factor or small set of factors) accurately distinguished between groups. Complicated algorithms constructed with cross-validation methods produced fair accuracy; complicated algorithms constructed with bootstrap optimism methods produced good accuracy, but multiple different algorithms with this method produced similar results.

**Conclusions:**

Findings were consistent with complex differences between people who engage in NSSI and suicide attempts. Specific complicated algorithms were either insufficient (cross-validation results) or unnecessary (bootstrap optimism results) to distinguish between these groups with high accuracy.

## Introduction

Nonsuicidal self-injury (NSSI) is defined as the direct and deliberate destruction of body tissue without any suicidal intent, whereas suicide attempt refers to the engagement in potentially self-injurious behavior with at least some intent to die from the behavior (1). Both behaviors are dangerous in nature, and both are unfortunately common. The prevalence rates of NSSI among the general population are estimated to be 17% among adolescents, 13% among young adults, and 5.5% among adults (2). For suicide attempt, the lifetime prevalence rates are estimated to be 2–4% (3, 4). Given that NSSI significantly increases risk for future suicide attempt (5) and suicide attempt is associated with worse treatment course and increased risk of mortality (6, 7), it is important to understand why certain individuals only engage in NSSI whereas others engage in suicide attempt. A first step toward answering this question is to understand how the characteristics of individuals engaging in NSSI and those engaging in suicide attempt differ cross-sectionally.

There are three general ways that individuals with NSSI and individuals with suicide attempt might differ (**Table 1**). First, they might differ in a *simple* way. That is, one or a small set of factors might be both necessary and sufficient to distinguish between them. One example of a simple difference is how atoms are different from each other: the number of protons is the necessary and sufficient factor to identify each type of atom. Importantly, simple differences entail easily comprehensible and sharp distinctions rather than oversimplification. In terms of the differences between individuals engaging in NSSI and suicide attempt, the interpersonal theory of suicide, one of the most widely known theories in the field, posits that the presence of both suicidal desire and acquired capability for suicide (*i.e.*, fearlessness about death) leads to suicidal behaviors (8, 9). Therefore, the key differentiating factors between individuals who only engage in NSSI and those who attempt suicide should be the combination of both suicidal desire and capability for suicide (8–10).

**Table 1:** Possible differences between individuals engaging in NSSI and suicide attempt.

	Simple	Complicated	Complex
**Definitions**	One or a small number of factors are both necessary and sufficient for accurate distinction.	A specific set of a large number of factors is both necessary and sufficient for accurate distinction.	Many (but not all) combinations of factors are sufficient for accurate distinction, but no combination is necessary.
**Examples**	The number of protons is both a necessary and sufficient factor to accurately distinguish between different types of atoms.	The presence of the following components is both necessary and sufficient to accurately distinguish between a functioning smartphone and a nonfunctioning smartphone: a circuit board, a speaker, a microphone, an antenna, a battery, a display screen, and a SIM card.	The solutions to the following mathematical problems are complex: *a + b = 1* *a + b + c + … + x + y + z = 1*
**NSSI and** **Suicide Attempt Examples**	The presence of suicidal desire and acquired capability for suicide might be both necessary and sufficient to distinguish between individuals only engaging in NSSI and individuals engaging in suicide attempt.	The presence of the following factors might be both necessary and sufficient to distinguish between individuals only engaging in NSSI and individuals engaging in suicide attempt: suicidal plans, nonzero suicidal desire, nonzero suicidal intent, acquired capability for suicide, no reasons for living, loneliness, hopelessness, access to means, and recent stressors.	One possible combination that might accurately distinguish between individuals only engaging in NSSI and individuals engaging in suicide attempt: above 60 years old + male + … + access to firearm = an individual engaging in suicide attempt Another possible combination: bullied + low socioeconomic status + childhood abuse + … + lack of friends = an individual engaging in suicide attempt One combination that might not distinguish the two groups: shoe size above five + yellow as favorite color + … + have a pet

Second, individuals engaging in NSSI and those engaging in suicide attempt might differ in a *complicated* manner, such that a specific set of a large number factors is both necessary and sufficient to accurately classify them. As an example of a complicated difference, a functioning smartphone requires a large number of working components, including a circuit board, a speaker, a microphone, an antenna, a battery, a display screen, and a SIM card. If any component in the specific set is missing (*e.g.*, a dead battery), the smartphone becomes nonfunctioning. That is, in order to distinguish between functioning and nonfunctioning smartphones, the above mentioned combination of a large number of factors is both necessary and sufficient. Any phones with all the above components present are considered functioning, and any phones with even just one component missing are considered nonfunctioning. Even though complicated differences involve a large number of factors, the distinctions are nonetheless sharp and clear.

In the context of NSSI and suicide attempt, perhaps individuals engaging in suicide attempt exhibit a specific set of characteristics that is both necessary and sufficient to distinguish the two groups. For example, all individuals with suicide attempt might have the following characteristics: presence of suicidal plans, nonzero suicidal desire, nonzero suicidal intent, acquired capability for suicide, no reasons for living, loneliness, hopelessness, access to means, and recent stressors. If the combination of these factors is both necessary and sufficient to distinguish between individuals with NSSI and those with suicide attempt, it entails that we could classify any individual with even one of the factors lacking as an individual with NSSI (*vs*. NSSI and suicide attempt) with a high degree of certainty. For individuals with all the factors present, we could confidently classify this individual as someone engaging in suicide attempt.

Third, *complex* differences might exist between the two groups. Colloquially, it is common to refer to complicated systems and differences as complex. For example, in our prior work we sometimes referred to complicated algorithms and complicated factor relationships as complex (11). But in the technical sense, there are many important differences between complicated systems/differences and complex systems/differences (12–16), highlighting the need to distinguish between complicated and complex. One such difference concerns necessary and sufficient factors. Whereas complicated systems/differences involve a combination of necessary and sufficient factors (see above), complex systems/differences do not. If the difference between two groups is complex, there may be no algorithm that is sufficient to distinguish between all members of the two groups (*i.e.*, no sufficient combination of factors). If a sufficient algorithm is found, the differences between the groups would still be complex if multiple algorithms with different factors or factor combinations were also sufficient to distinguish between the groups (*i.e.*, no necessary factors or factor combinations).

It is important to note that there are degrees of complexity. For example, an algorithm that correctly classified 70% of the members of two groups would indicate more complex differences than an algorithm that correctly classified group 95% of group members (*i.e.*, less sufficiency and, thus, greater complexity indicated by the 70% algorithm). Similarly, group differences would be considered more complex if 1,000 algorithms were sufficient to distinguish between groups than if only two algorithms were sufficient to distinguish between groups (*i.e.*, less necessity and, thus, greater complexity indicated in the scenario where 1,000 algorithms were sufficient).

**Figure 1:**
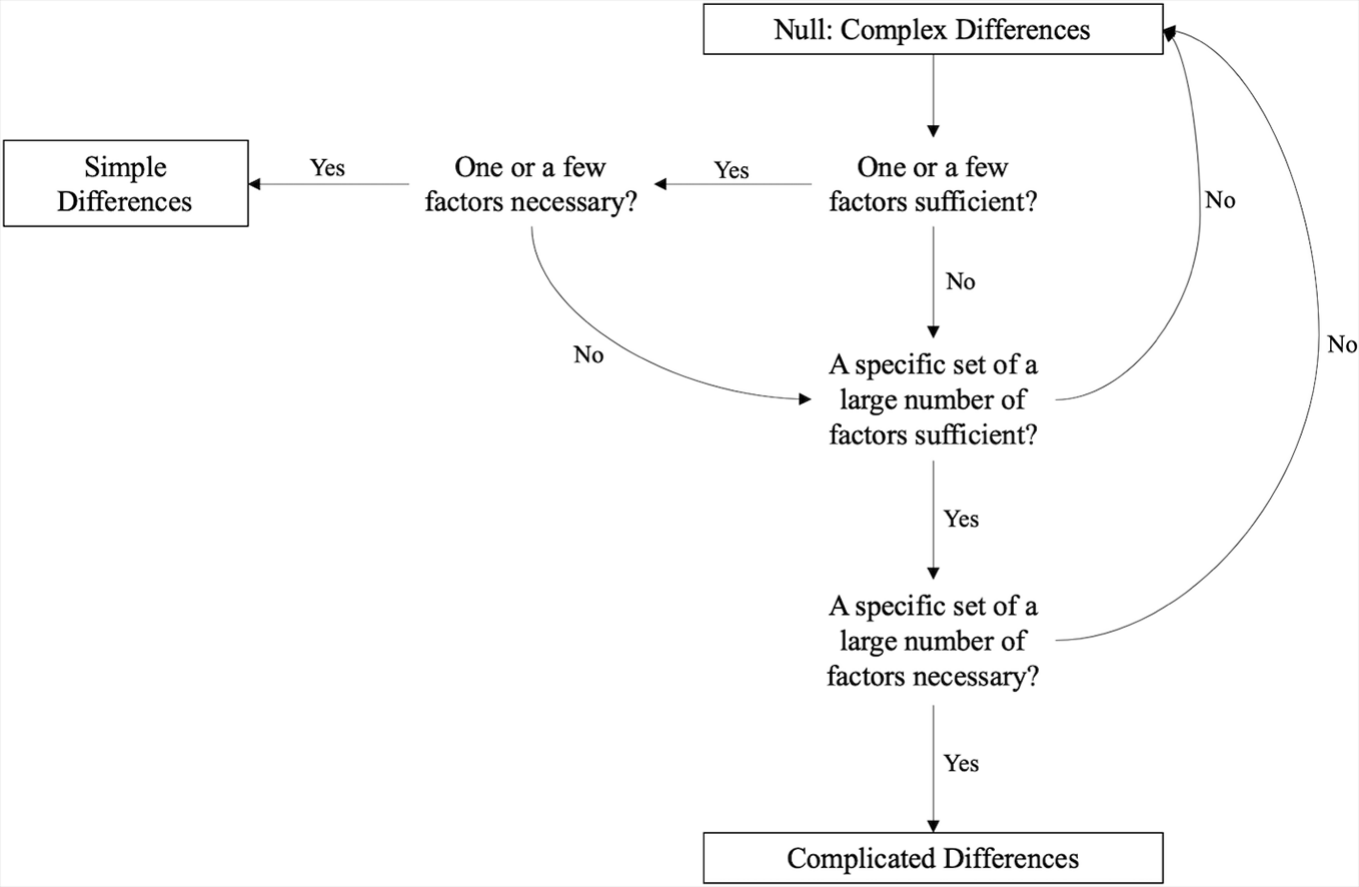
Evidence needed to constrain complex differences to simple or complicated differences. The null model is complexity, and evidence must be provided to constrain from a complex model to a complicated or simple model. Although sufficiency indicates perfect classification of the two groups, we lowered our criterion for sufficiency to good classification accuracy in terms of diagnostic accuracy metrics (*e.g.*, areas under the curve [AUCs] ~ 0.90) in consideration of measurement error. To demonstrate that one factor or one factor combination is necessary, it must be shown that no other algorithms with different factors or factor combinations are also sufficient (*i.e.*, yields good classification accuracy).

Although it is intuitive for humans to attempt to model systems as simple (17–19), most natural systems are complex (20–22). As such, many consider complexity to be the default model; evidence must be provided to constrain from a complex model to a complicated or simple model (**Figure 1**). To constrain from a complex model to a complicated model, evidence must be shown that a complicated combination of factors is both necessary and sufficient to distinguish between all members of two groups. To further constrain from a complicated to a simple model, evidence must be shown that a simple combination of factors is both necessary and sufficient to distinguish between all members of two groups. Several lines of evidence have led some researchers to suggest that most biological, psychological, and social phenomena are complex rather than complicated or simple (16, 20, 22–26). We likewise hypothesize that the differences between people who engage in NSSI and people who attempt suicide are complex rather than complicated or simple. We accordingly hypothesize that no simple or complicated algorithm will be necessary and sufficient to correctly distinguish between all (or nearly all) people who engage in NSSI and suicide attempts.

The present study will test this hypothesis by evaluating whether any simple or complicated algorithms are necessary and sufficient to distinguish between people who engage in NSSI and suicide attempts. In consideration of measurement error, we will lower our criterion for sufficiency from perfectly distinguishing between these two groups to distinguishing between these two groups with very good accuracy in terms of diagnostic accuracy metrics (*e.g.*, areas under the curve [AUCs] ~ 0.90). To test for simple differences, we will conduct univariate logistic regression analyses for each available factor. In addition, we will test a theoretically hypothesized simple difference by entering acquired capability for suicide, suicidal desire, and their interaction term as independent variables into a multiple logistic regression analysis (10). To support simple differences between individuals engaging in NSSI and suicide attempt, either the individual factors or the theoretically informed multiple logistic regression model should produce high classification accuracy. The absence of such evidence would suggest that these group differences are either complicated or complex.

To test for complicated differences, we will use multiple logistic regression analyses and machine learning analyses to construct complicated algorithms to distinguish between people who engage in NSSI and people who attempt suicide. To support complicated differences, two bars must be cleared: sufficiency and necessity. First, to clear the sufficiency bar, at least one algorithm must accurately distinguish between the two groups. The absence of such evidence would suggest that these group differences are complex. Second, if the sufficiency bar is cleared, to additionally clear the necessity bar, only one algorithm should accurately distinguish between the two groups. If more than one algorithm (*e.g.*, with different factors or a different combination of the same factors) produces high accuracy, this would violate necessity and indicate that group differences are complex.

The results of this study will advance the understanding of the nature of differences among individuals engaging in NSSI and suicide attempt, providing a foundation from which we can better understand why some people engage in NSSI whereas others engage in suicide attempts.

## Method

### Participants

A total of 954 participants were selected from a high-risk sample recruited internationally for a larger study (27). Participants were recruited from online forums that focused on topics of psychopathology, self-injury, and suicide. The inclusion criteria of the larger study required that participants must (a) be at least 18 years of age or older; (b) demonstrate sufficient English fluency to understand study instructions; (c) have engaged in nonsuicidal self-cutting at least twice in the past two weeks, have attempted suicide in the past year, or have thought about suicide more days than not in the past two weeks. The third inclusion criterion was designed to balance the need of recruiting a large sample to avoid potential model overfitting (see *Modeling Approach* below) and the need of recruiting a severe sample to ensure sufficient variance in the data (*e.g.*, a sufficient number of suicide attempts). Because self-cutting is a severe and yet common form of NSSI (28), the frequency of self-cutting was used as a screening criterion. Participants with other forms of NSSI (*e.g.*, self-burning) were not excluded if they met one of the three criteria on previous self-injurious thoughts and behaviors.

In addition to the inclusion criteria of the larger study, the present study required that participants must have either engaged in NSSI (N = 319) or attempted suicide (N = 635) in their lifetime. For participants who met the inclusion criteria of the original larger study because they had thought about suicide more days than not in the past two weeks at screening (but might not have engaged in nonsuicidal self-cutting at least twice in the past two weeks or attempted suicide in the past year), they were retained for the present study as long as they have engaged in NSSI or suicide attempt at least once in their lifetime.

Among the 954 participants, the mean age was 26.30 (*SD* = 7.11). More than half of the sample reported female gender (67.71%), with the rest reporting male gender (27.25%), other (3.78%), and prefer not to say (4.72%). The sample was predominantly White (79.67%), with the rest identifying as Black/African American (3.67%), Asian (5.87%), Hispanic or Latino (4.51%), Native American and Indigenous Peoples (0.84%), and other (5.45%). In terms of sexual orientation, 51.89% of the sample identified as heterosexual, while the remainder were bisexual (36.48%), homosexual (6.92%), or preferred not to disclose (4.72%).

### Procedures

The Institutional Review Boards at Florida State University and Vanderbilt University approved all study procedures. With the approval of online forum moderators, study advertisements were posted in web forums about mental health, self-injury, and suicide. Individuals interested in participation were asked to complete a brief screening survey to determine their eligibility. To ensure anonymity, individuals were asked to provide a non-identifiable email address at the end of the screening survey (*e.g.*, without names, date of birth, school and work information) for future study communication. Eligible individuals who provided consent were emailed their unique, randomly generated identification number and a link to complete the study assessment. The survey included approximately 50-min of computerized tasks and questionnaires. Within 24 h of completion, participants were provided with a $10 electronic Amazon gift card as study compensation.

The present study elected to collect data online due to the benefits of this method and at the same time implemented multiple procedures to guard against potential threats to validity. The advantages of online recruitment include easier access to diverse populations, minimal geographical constraints, and increased possibility of recruiting severe clinical samples (29). In addition, research has shown that online studies produce comparable results to the traditional face-to-face settings (30). Consistent with best practices of online recruitment (31, 32), multiple steps were adopted during the screening process to ensure data quality. First, to reduce the likelihood of individuals intentionally altering their responses to gain access to the study, the inclusion criteria were not included in the study advertisements, and relevant screening questions were embedded among irrelevant filler questions. Second, duplicate items and free-response items were included in the screening survey to check for consistency and English fluency. Third, to prevent the same individuals from entering the study more than once, only unique IP addresses were allowed to participate in the study.

### Measures

We included factors that have been found to be broadly associated with NSSI and suicide attempt (33, 34), such as demographics, psychopathology, prior self-injurious thoughts and behaviors, and explicit and implicit processes. We intentionally balanced relatively stable, distal factors with more variant and proximal factors (*e.g.*, affective states). Theoretically relevant constructs (*e.g.*, hopelessness, capability for suicide) were also assessed. Given that hundreds of factors have been studied in relation to NSSI and suicide attempt, it was not feasible to include all possibly relevant factors. However, the potential omission of one or a few specific factors is unlikely to impact the results. Meta-analytic evidence suggests that hundreds of factors confer risk for NSSI and suicide attempt to a similar extent, and no factor exerts particularly strong effects (33, 34). Therefore, it is unlikely that any factors not included in the present study would exert an effect above and beyond the included factors.

#### Demographics

Demographic information including age, employment, gender, sexual orientation, and race was assessed using brief self-report items.

#### Modified Suicidal Thoughts and Behaviors Interview (SITBI)

The SITBI (35) is a standardized and validated measure assessing for thoughts of NSSI, NSSI, suicidal thoughts, plans, preparations, and attempts. The interview appears valid as it has been shown to strongly correspond to other measures of suicidal thoughts, suicide attempt, and NSSI. The scale also demonstrates strong interrater reliability and test-retest reliability (35). The present study adopted the modified SITBI, a self-report adaptation of the original interview that has been used in previous studies (36, 37). In this study, the modules on NSSI and suicidal plans, preparations, and attempts were administered.

#### Acquired Capability for Suicide Scale-Fearlessness about Death (ACSS-FAD)

The seven-item ACSS-FAD (38) measures fearlessness about death, an important construct theorized to distinguish between individuals who engage in NSSI and suicide attempts (38). Participants were asked to rate on a four-point Likert scale from 0 (not at all like me) to 4 (very much like me) on statements such as “I am very much afraid to die.” Higher scores suggest greater capability for suicide. This measure has been shown to demonstrate good convergent and discriminant validity (38). The internal consistency of the scale was good (Cronbach's *α* =.85).

#### Affective States Questionnaire (ASQ) 

The ASQ (39, 40) was included to assess nine different negative affective states, such as feelings of self-hatred, abandonment, and humiliation. Participants were asked to answer either “yes” or “no” to experiences of these negative states. The ASQ demonstrates good validity and is predictive of future suicidal behavior (39).

#### Modified Affect Misattribution Procedure (AMP)

The present study included the modified AMP (41–43) to assess implicit affect toward suicide and self-injury stimuli given that prior studies have established that reduced implicit aversion toward suicide and self-injury stimuli are associated with increased risk for NSSI (41, 44). On each trial of the AMP, an image was presented to the participants on the computer screen. Subsequently, an ambiguous Chinese symbol was presented. Participants were told to ignore the first image stimuli and treat them only as cues that the Chinese symbols were about to flash on the screen. Participants were asked to rate whether they found the Chinese symbols to be pleasant or unpleasant. Research has shown that the pleasantness of the image or word stimuli influences the ratings of the subsequent Chinese symbols (43). Through this misattribution, participants' implicit affective reactions to the original stimuli were assessed. For the present study, we used both positive stimuli (*e.g.*, images of pets, babies, beaches) and suicide/self-injury stimuli. The intensity of suicide/self-injury stimuli ranged from low (*e.g.*, pills, heights, body bags), moderate (*e.g.*, a floating body in the water, bleeding from self-cutting), to high (*e.g.*, body with severe burn, corpse with fatal gunshot wound to head). The internal consistency was good for each category of images: Cronbach's *α* was.85 for both the low-intensity and moderate-intensity suicide/self-injury images,.86 for the high-intensity suicide/self-injury images, and.80 for the positive images.

#### Beck Scale for Suicide Ideation (BSS)

The 21-item BSS (45, 46) measures suicidal thoughts and behaviors. In this study, items 1–5 on suicidal desire were administered. Each item was rated on a Likert scale ranging from 0 to 2, with lower scores indicating lower desire for suicide. The internal consistency for the suicidal desire subscale was acceptable (*α* =.85).

#### Brief Agitation Measure (BAM)

The BAM (47) includes three self-report items assessing for agitation in the past week. Participants were asked to rate each statement (*e.g.*, “I want to crawl out of my skin”) on a seven-point Likert scale ranging from 0 (strongly disagree) to 6 (strongly agree). Higher scores indicate higher levels of agitation. This scale has been shown to have good validity and reliability (47). Cronbach's *α* indicated good internal consistency (*α* =.84).

#### Brief Symptom Inventory-18 (BSI-18)The BSI-18

The BSI-18 (48) was adopted to inquire past week psychological symptoms (*e.g.*, anhedonia, pains in heart or chest, nausea). Participants rated how much they experienced each symptom on a five-point Likert scale from 0 (not at all) to 4 (extremely). Higher scores reflect greater psychological distress. Previous research has found that this scale has good reliability and validity. The internal consistency of this scale was good (*α* =.81).

#### Explicit Affective Ratings

In addition to measuring implicit affects, we also measured explicit affects (41, 49) toward positive, and suicide and self-injury stimuli given that implicit and explicit associations tend to diverge under certain circumstances [*e.g.*, motivation to disguise explicit attitudes; (50, 51)]. Moreover, reduced explicit aversion to suicide and self-injury stimuli has been linked with increased risk for NSSI (41, 42). Explicit affect was assessed using a 10-point Likert scale. Higher scores indicated that participants found the stimuli more pleasant. Five positive images (Cronbach's *α* =.79) and five suicide/self-injury images (Cronbach's *α* =.90) were drawn from the stimuli used in the AMP task described previously for the present assessment. For suicide/self-injury stimuli, the images were of moderate intensity.

#### Disgust With Life Scale (DWLS)

With 12 self-report items, the DWLS (52, 53) includes two subscales (*i.e.*, disgust with self, disgust with others). Participants rated each item (*e.g.*, “I am disgusted with myself”) on a seven-point Likert scale from 0 (not at all true of me) to 6 (very much true of me). Higher scores on the DWLS indicate greater disgust toward self and others. The subscales have shown strong convergent validity with other measures of disgust (52), as well as good internal consistency (*α* =.90).

#### Insomnia Severity Index (ISI)

The ISI (54) is a seven-item self-report inventory that measures symptoms of insomnia. The index has shown adequate internal reliability and convergent validity (55). The internal consistency of this scale was good (*α* =.86).

### Statistical Analyses

#### Missing Data

A total of 33 factors were considered (see **Tables 2** and **3** for details). Missing data were minimal (< 0.01%) and addressed using multiple imputation. No outcome data (*i.e.*, engagement in NSSI or suicide attempt) were missing.

**Table 2:** Univariate logistic regression analyses based on 10-fold cross-validation.

Variables	AUC	95% CI	Precision	Recall	Brier
ACSS-FAD	0.57	[0.46, 0.67]	0.72	0.62	0.43
Age	0.54	[0.44, 0.64]	0.72	0.59	0.50
AMP—Positive	0.52	[0.42, 0.63]	0.69	0.75	0.45
AMP —Suicide/Self-Injury					
Low Intensity	0.53	[0.42, 0.64]	0.70	0.62	0.49
Moderate Intensity	0.53	[0.43, 0.64]	0.70	0.67	0.48
High Intensity	0.54	[0.43, 0.64]	0.70	0.63	0.47
ASQ—Abandonment	0.54	[0.44, 0.65]	0.71	0.57	0.49
ASQ—Anxiety	0.51	[0.45, 0.58]	0.68	0.95	0.35
ASQ—Desperation	0.55	[0.45, 0.65]	0.70	0.76	0.40
ASQ—Guilt	0.52	[0.41, 0.62]	0.69	0.74	0.47
ASQ —Hope	0.48	[0.38, 0.57]	0.67	0.92	0.47
ASQ—Humiliation	0.53	[0.43, 0.63]	0.72	0.49	0.53
ASQ—Loneliness	0.48	[0.39, 0.56]	0.68	0.75	0.57
ASQ—Rage	0.52	[0.41, 0.62]	0.69	0.76	0.47
ASQ—Self-Hate	0.52	[0.43, 0.61]	0.68	0.88	0.38
BAM	0.56	[0.45, 0.66]	0.71	0.64	0.44
BSI	0.58	[0.48, 0.69]	0.74	0.59	0.42
DWLS—Other	0.55	[0.44, 0.66]	0.71	0.65	0.44
DWLS—Self	0.58	[0.47, 0.69]	0.73	0.63	0.41
Employment	0.53	[0.43, 0.63]	0.71	0.62	0.49
Explicit Ratings—Positive	0.54	[0.43, 0.64]	0.71	0.59	0.50
Explicit Ratings—Suicide/Self-Injury	0.54	[0.44, 0.65]	0.71	0.59	0.48
Gender	0.46	[0.37, 0.55]	0.67	0.97	0.55
ISI	0.59	[0.48, 0.69]	0.74	0.59	0.42
Preparations for Suicide	0.67	[0.58, 0.77]	0.77	0.90	0.25
Confidence in Killing Self during Preparations	0.73	[0.64, 0.83]	0.84	0.77	0.25
Race	0.51	[0.43, 0.59]	0.73	0.46	0.59
Sexual Orientation	0.50	[0.42, 0.58]	0.72	0.56	0.57
Suicidal desire (BSS)	0.57	[0.47, 0.68]	0.74	0.53	0.45
Suicide Plans	0.53	[0.48, 0.58]	0.68	0.99	0.32
Past Month Frequency	0.58	[0.48, 0.68]	0.76	0.45	0.47
Intent on Acting on Plans	0.67	[0.57, 0.77]	0.80	0.71	0.32
Likelihood of Future Plans	0.57	[0.47, 0.67]	0.72	0.65	0.42

AUC, area under the receiver operating characteristic curve; AUCs of 0.50, chance-level discriminative accuracy; AUCs of 1.0, perfect discriminative accuracy; CI, Confidence Interval; precision, positive predictive value; recall, sensitivity; precision and recall both range from 0 to 1; with higher values indicating better model performance; Brier scores of 0, perfect calibration; with scores closer to 0 indicating better calibration; ACSS, Acquired Capability for Suicide Scale – Fearlessness about Death; AMP, Affect Misattribution Procedure; ASQ, Affective State Questionnaire; BAM, Brief Agitation Measure; BSI, Brief Symptom Inventory; DSWS, Disgust with Self and World Scale; ISI, Insomnia Severity Index; BSS, Beck Scale for Suicidal Ideation.

**Table 3:** Univariate logistic regression analyses based on bootstrap optimism correction.

Variables	AUC	95% CI	Precision	Recall	Brier
ACSS-FAD	0.56	[0.53, 0.60]	0.72	0.58	0.43
Age	0.53	[0.50, 0.56]	0.71	0.44	0.51
AMP—Positive	0.52	[0.48, 0.55]	0.68	0.74	0.47
AMP—Suicide/Self-Injury					
Low Intensity	0.52	[0.48, 0.55]	0.68	0.71	0.51
Moderate Intensity	0.51	[0.48, 0.55]	0.68	0.74	0.48
High Intensity	0.53	[0.49, 0.56]	0.69	0.60	0.47
ASQ—Abandonment	0.53	[0.49, 0.56]	0.69	0.58	0.50
ASQ—Anxiety	0.51	[0.49, 0.53]	0.67	0.95	0.37
ASQ—Desperation	0.55	[0.52, 0.58]	0.70	0.72	0.40
ASQ—Guilt	0.51	[0.47, 0.54]	0.67	0.79	0.48
ASQ—Hope	0.50	[0.47, 0.53]	0.66	0.91	0.43
ASQ—Humiliation	0.53	[0.49, 0.56]	0.70	0.52	0.54
ASQ—Loneliness	0.50	[0.47, 0.52]	0.66	0.73	0.61
ASQ—Rage	0.50	[0.47, 0.54]	0.67	0.81	0.47
ASQ—Self-Hate	0.52	[0.49, 0.55]	0.68	0.88	0.39
BAM	0.55	[0.51, 0.58]	0.70	0.60	0.45
BSI^b^	0.58	[0.55, 0.62]	0.74	0.56	0.42
DWLS—Other	0.55	[0.52, 0.58]	0.71	0.56	0.45
DWLS—Self^a^	0.58	[0.55, 0.61]	0.73	0.60	0.41
Employment	0.53	[0.50, 0.57]	0.71	0.59	0.50
Explicit Ratings—Positive	0.52	[0.48, 0.55]	0.68	0.57	0.52
Explicit Ratings—Suicide/Self-Injury	0.53	[0.49, 0.56]	0.69	0.50	0.49
Gender	0.49	[0.48, 0.51]	0.67	0.71	0.67
ISI^b^	0.59	[0.55, 0.62]	0.74	0.56	0.42
Preparations for Suicide^a,b^	0.68	[0.65, 0.71]	0.77	0.90	0.25
Confidence in Killing Self during Preparations^a,b^	0.73	[0.70, 0.76]	0.83	0.78	0.25
Race	0.52	[0.49, 0.54]	0.70	0.40	0.59
Sexual Orientation	0.51	[0.49, 0.53]	0.74	0.45	0.62
Suicidal desire (BSS)	0.57	[0.54, 0.60]	0.73	0.53	0.45
Suicide Plans	0.53	[0.52, 0.55]	0.68	0.99	0.33
Past Month Frequency^b^	0.58	[0.55, 0.61]	0.76	0.50	0.47
Intent on Acting on Plans^a,b^	0.67	[0.64, 0.70]	0.79	0.69	0.30
Likelihood of Future Plans^a^	0.57	[0.54, 0.60]	0.72	0.60	0.40

AUC, area under the receiver operating characteristic curve; AUCs of 0.50, chance-level discriminative accuracy; AUCs of 1.0, perfect discriminative accuracy; CI, Confidence Interval; precision, positive predictive value; recall, sensitivity; precision and recall both range from 0 to 1; with higher values indicating better model performance; Brier scores of 0, perfect calibration; with scores closer to 0 indicating better calibration; ACSS, Acquired Capability for Suicide Scale – Fearlessness about Death; AMP, Affect Misattribution Procedure; ASQ, Affective State Questionnaire; BAM, Brief Agitation Measure; BSI, Brief Symptom Inventory; DSWS, Disgust with Self and World Scale; ISI, Insomnia Severity Index; BSS, Beck Scale for Suicidal Ideation; Superscript a indicates the top five variables in the random forest algorithms that yielded the highest mean decrease in accuracy; Superscript b indicates the five most discriminative variables according to the univariate analyses.

#### Modeling Approach

Considering that it is common for individuals to engage in both NSSI and suicide attempt (56, 57), we elected *not* to exclude individuals with both behaviors from the models. Individuals engaging in both NSSI and suicide attempt were grouped with individuals with suicide attempt only. That is, the models were tasked with separating individuals with suicide attempt (regardless of their engagement in NSSI) from individuals engaging in NSSI only. This decision was intended to increase the clinical relevance of the study as many clinicians are concerned with whether patients might engage in suicide attempt. Retaining the whole sample would also allow the models to leverage a larger sample size and thereby producing more precise model performance estimates (*i.e.*, narrower confidence intervals). For completeness, we repeated analyses based on the subsample of individuals with NSSI only and individuals with suicide attempt only; the results were statistically identical (**Tables 4** and **5**).

**Table 4:** Model performance based on 10-fold cross-validation.

	Simple Differences		Complicated Differences	
	Test for Sufficiency		Test for Sufficiency	
	Univariate LR	Theoretically Informed Model	Multiple LR	Random Forests
	Average	Suicidal Desire & Capability for Suicide	All Variables	All Variables
**Entire Sample (NSSI Only: N = 319; Suicide Attempt with or without NSSI: N = 635)**				
AUC	0.55 [0.45, 0.65]	0.58 [0.48, 0.69]	0.73 [0.63, 0.82]	0.72 [0.63, 0.82]
Precision	0.72	0.74	0.84	0.84
Recall	0.68	0.58	0.75	0.73
Brier	0.45	0.42	0.27	0.28
**Subsample (NSSI Only: N = 319; Suicide Attempt without NSSI: N = 52)**				
AUC	0.57 [0.36, 0.78]	0.60 [0.38, 0.81]	0.70 [0.48, 0.91]	0.74 [0.54, 0.93]
Precision	0.19	0.20	0.34	0.30
Recall	0.74	0.76	0.62	0.77
Brier	0.45	0.45	0.23	0.29

AUC, area under the receiver operating characteristic curve; AUCs of 0.50, chance-level discriminative accuracy; AUCs of 1.0, perfect discriminative accuracy; precision, positive predictive value; recall, sensitivity; precision and recall both range from 0 to 1; with higher values indicating better model performance; Brier scores of 0, perfect calibration; with scores closer to 0 indicating better calibration; LR, logistic regression.

**Table 5:** Model Performance Based on Bootstrap Optimism Correction.

	Simple Differences		Complicated Differences				
	Test for Sufficiency		Test for Sufficiency		Test for Necessity		
	Univariate LR	Theoretically Informed Model	Multiple LR	Random Forests	Random Forests	Random Forests	Random Forests
	Average	Suicidal Desire & Capability for Suicide	All Variables	All Variables	Without 5 Most Important Variables	Without 5 Most Discriminative Variables	Without 10% Randomly Selected Variables
**Entire Sample (NSSI Only: N = 319; Suicide Attempt with or without NSSI: N = 635)**							
AUC	0.55 [0.52, 0.58]	0.58 [0.54, 0.61]	0.73 [0.70, 0.76]	0.89 [0.87, 0.91]	0.84 [0.81, 0.86]	0.84 [0.81, 0.86]	0.89 [0.87, 0.91]
Precision	0.71	0.73	0.84	0.91	0.87	0.87	0.91
Recall	0.66	0.56	0.76	0.96	0.97	0.97	0.96
Brier	0.46	0.43	0.26	0.09	0.12	0.12	0.09
**Subsample (NSSI Only: N = 319; Suicide Attempt without NSSI: N = 52)**									
AUC	0.54 [0.47, 0.61]	0.57 [0.50, 0.64]	0.76 [0.69, 0.82]	0.84 [0.77, 0.90]	0.81 [0.75, 0.88]	0.81 [0.75, 0.88]	0.83 [0.77, 0.90]
Precision	0.17	0.18	0.35	0.97	0.99	0.98	0.96
Recall	0.68	0.62	0.74	0.68	0.63	0.63	0.67
Brier	0.46	0.46	0.22	0.05	0.05	0.05	0.05

AUC, area under the receiver operating characteristic curve; AUCs of 0.50, chance-level discriminative accuracy; AUCs of 1.0, perfect discriminative accuracy; precision, positive predictive value; recall, sensitivity; precision and recall both range from 0 to 1; with higher values indicating better model performance; Brier scores of 0, perfect calibration; with scores closer to 0 indicating better calibration; LR, logistic regression.

All statistical analyses were performed in R (58) *via glm* in base R, and *randomForest* and *pROC* packages. To test for simple differences, univariate analyses were conducted for each factor. Even though the primary aim of the study is not to test specific theories, we also considered suicidal desire and capability for suicide as an example of theorized simple difference given that the interpersonal theory (8, 9) is one of the most prominent theories in the field that also has well-established measures on the posited factors. To test this theoretically-driven model, a multiple logistic regression model with suicidal desire (as measured by BSS), acquired capability for suicide (as measured by ACSS-FAD), and their interaction term as independent variables was conducted.

A range of analyses were conducted to test for complicated differences between individuals engaging in NSSI and suicide attempt (*i.e.*, to constrain from a complex model to a complicated model). We first examined whether multiple logistic regression analyses with all variables might be sufficient (again, operationalized as AUC ~.90 in consideration of measurement errors). This decision was based on prior research supporting the utility of adopting logistic regression models in the classification and prediction of self-injurious thoughts and behaviors (59, 60). Second, we analyzed whether machine learning analyses might be sufficient in distinguishing the two groups. Specifically, we adopted random forest algorithms given that they have been commonly used in the field of suicide, self-injury, and medicine (61–64). As a nonparametric method, random forests might also serve as a complement to the multiple logistic regression model (see below for details). If neither model was sufficient in classifying individuals with NSSI and suicide attempt, it would indicate that the differences between the two groups were likely complex instead of complicated. If either model cleared the sufficiency requirement for complicated differences, we would then test for necessity by dropping variables included in the models in various ways (*i.e.*, removing the top five most important factors identified by random forests, the top five most discriminative factors identified by univariate analyses, and a randomly selected 10% of the variables). If results showed that multiple models could produce similarly accurate classification, they would suggest that none of the models was necessary. This would again indicate complex differences.

#### Random Forests

Given that random forest algorithms are relatively new compared to traditional logistic regression methods, we hereby provide a brief overview of this method. The random forest algorithm consists of an ensemble of decision trees. Randomness was strategically introduced into the algorithm to avoid overfitting (*i.e.*, overcapitalizing on noise within the present sample) and to increase the likelihood that the algorithm would generalize to a different dataset. For instance, within each tree in the ensemble, only a random subset of factors is allowed to be considered at each “split” of the decision tree. This procedure results in trees that are less correlated, thereby making the overall algorithm more reliable and robust. Per common practice in the field, the number of factors randomly considered at each split in this study was set as the square root of the total number of factors (65). The fitting process was repeated 500 times in this study to produce a forest of trees (66, 67). The outcome of the algorithm for each participant (*i.e.*, whether an individual engages in NSSI or suicide attempt) was determined by a majority vote from the 500 trees. The random forest algorithm also provides estimates of the importance of factors within the algorithm by averaging and standardizing the decrease in classification accuracy after randomly permuting each variable.

#### Internal Validation

Internal validation methods help to reduce overfitting, where algorithms may capitalize on noise in a given dataset, providing an estimate that may not generalize to a new dataset. We first employed 10-fold cross-validation, a commonly used internal validation method (65). This approach involves randomly dividing the data into 10 sets, where models are developed on the combination of nine sets and tested on the one selected set. This procedure is repeated 10 times, each time with a different set selected as the test set. Because of sample imbalance and the accompanied possibility that one set might not contain at least one individual with nonsuicidal self-injury to allow for validation, we adopted a stratified approach during the data splitting process.

We also employed bootstrap optimism correction as an additional internal validation technique. To implement this method, the model first needs to be trained on the complete available data, then on a set of bootstrap replicates created from the original data. One hundred replicates were generated in this study. The models built on the replicates are subsequently applied to the original data, yielding performance estimates called “out of bag” estimates. The mean difference between the bootstrapped performance estimates and the “out of bag” estimates represents the extent of overfitting, which is termed “optimism.” The model performance indices corrected for optimism can be obtained by subtracting the optimism from the original model performance indices.

Bootstrap optimism correction has been employed in prior work using machine learning to study NSSI and suicide attempts (62, 64). Some studies have indicated its particular appropriateness for small samples as this method allows training on the entirety of the data (68–71). However, recent work indicates that this approach may not adequately reduce overfitting in some cases, resulting in higher accuracy estimates than those obtained with other approaches (72). On balance, some studies indicate that bootstrap optimism correction methods perform similarly to other internal validation methods (73, 74), random forest models can generalize well to new data (75, 76), and random forest combined with bootstrap optimism correction performs similarly to other internal validation methods and other machine learning techniques (73, 77, 78). There is also evidence that Walsh et al.'s algorithm (64) using this approach generalizes well to new samples and new suicide-related outcomes (79, 80). Nonetheless, much remains unknown about how various methods perform under various conditions, so at a minimum these discrepancies indicate that it would be prudent to conduct analyses with multiple internal validation techniques.

#### Model Fit Indices

Consistent with prior research (64, 81), a range of model fit indices were adopted to evaluate model performance. Area Under the Receiving Operating Characteristic Curve (AUC) was used to assess the overall classification accuracy. Because individuals engaging in suicide attempt substantially outnumbered those only engaging in NSSI in the present sample, solely relying on AUC to evaluate models could be misleading. For instance, a model classifying everyone as engaging in suicide attempt might produce high AUC, but is not clinically meaningful. Therefore, we also considered indices such as precision (*i.e.*, positive predictive value) and recall (*i.e.*, sensitivity). Following guidelines in the field (34, 64, 81), AUCs of 0.50 to 0.59 suggest extremely poor classification, 0.60 to 0.69 poor classification, 0.70 to 0.79 fair classification, 0.80 to 0.89 good classification, and above.90 excellent classification. These guidelines were also applied to precision and recall.

Additionally, Brier score as a calibration index was considered. In the field of clinical psychology, discrimination indices (*e.g.*, AUC, precision, recall) have been more commonly used than calibration indices (82). Yet, in order for a model to be clinically useful, the probability of an outcome as estimated by the model should approximate the actual probability of such an event. In the context of this study, the proportion of individuals identified as engaging in suicide attempt compared to those identified as engaging in NSSI only by the model should match the actual proportion in the sample. A Brier score ranges from zero to one, with zero indicating a complete match between projected probability and the real-world probability. Higher scores indicate poorer model performance due to more deviation of the projected outcome probability from the real-world probability. Brier scores can be calculated with the following formula, $Brier=\frac{1}{N}\sum_{i=1}^{N}{\left(p_{i}-o_{i}\right)}^{2},$ where N is the sample size of classified individuals, p_i_ is the projected outcome for individual i, and o_i_ is the observed outcome (83).

## Results

### Baseline Characteristics

Among the 319 individuals engaging in NSSI but not suicide attempt, 90.91% endorsed self-cutting, 42.63% endorsed self-burning, and 61.76% endorsed using methods other than cutting and burning. Many of the individuals were still actively engaging in these behaviors at the time of the study. About 46.08% of participants reported having cut themselves within the past month, and 23.51% reported having done so within the past week. Approximately 5.96 and 3.45% of the participants reported having burned themselves in the past month and in the past week, respectively. In terms of using other NSSI methods, 28.53% reported such behaviors in the past month and 17.24% in the past week. Based on responses on the SITBI-SF (35), approximately 40.12% of the participants reported no desire to stop engaging in NSSI. In terms of self-rated estimated likelihood of engaging in NSSI again in the future, 94.36% reported nonzero likelihood, and 74.29% reported at least moderate likelihood (*i.e.*, at least 5 on a 0-to-10 Likert scale).

Among the 635 individuals with suicide attempt, the majority of the participants (91.65%) also endorsed previous engagement in NSSI. Most participants (75.59%) had attempted more than once in their lifetime. The median lifetime frequency of suicide attempts is 3 (*M* = 6.30, *SD* = 13.50). About 45.98% attempted in the past year, 13.23% in the past month, and 4.41% in the past week. Half of the participants (55.75%) reported at least one instance of attempt that resulted in at least moderate physical damage and required medical attention. According to responses on the SITBI-SF (35), 94.80% of the participants with lifetime history of suicide attempt noted nonzero likelihood to attempt suicide again in the future, with 66.30% noting at least moderate likelihood (*i.e.*, at least 5 on a 0-to-10 Likert scale).

### Model Performance

In terms of the possibility of simple differences between individuals engaging in NSSI and suicide attempt, univariate logistic regression analyses with both internal validation techniques showed that on average individual factors produced chance level classification accuracy, and that all factors produced AUCs lower than 0.75 (**Tables 2** and **3**). Univariate classification was weak across other metrics (*i.e.*, precision, recall, and Brier score) for most variables (**Tables 4** and **5**). The theoretically informed multiple logistic regression model including acquired capability for suicide, suicidal desire, and their interaction term produced near chance level accuracy, with fair precision, poor recall, and poor calibration (**Tables 4** and **5**). Thus, neither univariate models nor the theoretically informed models appeared sufficient for distinguishing between the two groups.

Regarding possible complicated differences, traditional multiple logistic regression with either internal validation technique yielded fair accuracy and did not appear sufficient in distinguishing individuals with NSSI and suicide attempt (**Tables 4** and **5**). That is, results from the multiple logistic regression analyses were unable to constrain from complex differences to complicated differences. When internally validated with 10-fold cross-validation, the random forest algorithm with all variables did not appear sufficient as it yielded only fair accuracy (**Table 4**). When internally validated with the bootstrap optimism correction method, the random forest algorithm with all variables yielded AUC close to.90, suggesting that it was sufficient in distinguishing the two groups (**Table 5**). The following variables were then removed from inclusion of the models: the top five most important variables (*i.e.*, confidence in killing self during preparations for suicide, intent on acting on suicide plans, lifetime history of preparations for suicide, self-rated likelihood of developing future suicide plans, disgust with self), the five most discriminative variables identified by univariate analyses (*i.e.*, confidence in killing self during preparations for suicide, lifetime history of preparations for suicide, intent on acting on suicide plans, insomnia, past month frequency of suicide plan), and a randomly selected 10% of variables. After removing variables in various ways, however, the algorithms produced similarly sufficient classifications (**Table 5**), indicating that none of the algorithms were necessary. In other words, results from random forests with either internal validation technique failed to constrain from complex differences to complicated differences. Result remained consistent when analyses were restricted to the sample of individuals with NSSI only and suicide attempt only (**Table 5**): no model was able to constrain complex differences to either simple or complicated differences.

## Discussion

Although researchers have long been interested in how people who engage in NSSI differ from people who engage in suicide attempts, the nature of these differences has remained unclear. The present findings indicated that these differences are complex in nature: results were unable to detect evidence of simple or complicated differences. Across all available variables considered in the study, no specific factor accurately separated the two groups in univariate analyses. The theoretically informed model with two factors (*i.e.*, acquired capability for suicide and suicidal desire) yielded chance level accuracy as well. These results suggest that it is unlikely for an individual factor or a small set of individual factors to be both necessary and sufficient to distinguish between individuals engaging in NSSI and suicide attempt. Multiple logistic regression analyses and random forest analyses with 10-fold cross-validation produced fair accuracy, indicating that complicated algorithms constructed with these methods were insufficient to distinguish between NSSI and suicide attempt groups with high accuracy. Random forest analyses with bootstrap optimism correction was sufficient to distinguish between these groups with high accuracy, but many complicated algorithms constructed with this approach produced comparable results. Accordingly, none of these algorithms was necessary to distinguish between these groups with high accuracy. These findings are most consistent with complex differences between people who engage in NSSI and people who attempt suicide, where no factor or factor combination is necessary and sufficient to distinguish between these groups.

The current findings are consistent with prior research on self-injurious thoughts and behaviors. Multiple meta-analyses examining predictors of NSSI and suicidal thoughts and behaviors have found that, on average, univariate predictions yielded accuracy only slightly above chance levels (33, 34). Such findings indicate that all known factors and simple combinations of factors are insufficient to accurately predict self-injurious thoughts and behaviors or to distinguish among subgroups of people who engage in self-injurious thoughts and behaviors. Also consistent with the present findings, several studies have found that complicated algorithms can produce fair-to-good accuracy using a range of statistical methods (64, 84–87). Among complicated algorithms that have produced highly accurate classification or prediction, evidence across (and sometimes within) studies indicates that no particular factor combination is necessary to produce high accuracy. These broader findings, along with the present findings, show that even complicated algorithms are either insufficient or unnecessary to produce high accuracy prediction or classification of self-injurious thoughts and behaviors. That is, existing evidence does not yet allow us to constrain from a complex view to a complicated view of self-injurious thoughts and behaviors.

All approaches employed in the present study converged on the same conclusion – that the differences among people who engage in NSSI and suicide attempts are complex. But the different approaches indicated different degrees of complexity. Multiple logistic regression and random forest with 10-fold cross-validation indicated a higher degree of complexity, as these complicated algorithms were neither sufficient nor necessary for high accuracy classification. Random forests with bootstrap optimism correction indicated a lower degree of complexity, with complicated algorithms that were sufficient but not necessary to produce high accuracy classification.

It is important to note that, so far in this paper, we have discussed *sufficiency* in terms of the ability to produce high accuracy classification within a single sample. However, NSSI and suicide research are primarily concerned with identifying simple or complicated factor combinations that accurately classify (or predict or cause) these phenomena across *all* samples. That is, we are primarily concerned with identifying nomothetic explanations or algorithms. To truly justify constraining from a complex to a complicated view of self-injurious thoughts and behaviors, we must show that a given algorithm is both sufficient and necessary across a large number of samples (ideally across different ages, cultures, *etc.*). Existing studies, including the present study, have been unable to detect a necessary and sufficient algorithm within a single sample, raising serious doubts about detecting such an algorithm that applies across all or most samples. It will always be possible that such a simple or complicated algorithm will be found but, in our opinions, this possibility no longer appears plausible. We believe that it is most plausible that the causes, predictors, and correlates of self-injurious thoughts and behaviors are complex, and that it is most useful for researchers and clinicians to assume this complexity.

So, what would it mean if the causes, predictors, and correlates of self-injurious thoughts and behaviors truly are complex? In our opinions, this would mean at least six things. First, self-injurious thoughts and behaviors work like most other psychological phenomena, which are complex on the level of biopsychosocial factors [*e.g.*, emotions: see (23, 24)]. Second, the causes, predictors, and correlates of self-injurious thoughts and behaviors are indeterminate (*i.e.*, show *degeneracy* and *pluripotentiality*, which are core feature of complex systems), but they are not random. There are likely to be many notable regularities across instances of self-injurious thoughts and behaviors (*e.g.*, negative affect). But these regularities are unlikely to be either sensitive or specific to self-injurious thoughts and behaviors, and there are likely to be many irregularities. Third, this indeterminacy will likely make it impossible to form a simple (or even complicated) theory of self-injurious thought and behavior causes that accounts for most instances. Fourth, this indeterminacy likely places a ceiling effect on the accuracy of prediction algorithms, especially across samples. Fifth, this indeterminacy likely places a ceiling effect on intervention efficacy, especially for interventions that target a few specific factors. Indeed, Fox et al. (88) meta-analyzed over 300 randomized controlled trials for self-injurious thoughts and behavior, finding that many interventions slightly reduce these phenomena (~8–15% reductions), but no intervention produces large or even moderate reductions. Sixth, self-injurious thought and behavior research may benefit from moving to a different level of analysis. Although the contributions to these phenomena may be complex on the level of biopsychosocial factors, they may not be complex on other levels. Facing similar difficulties, researchers in other areas of psychological science—most notably affective science (23, 89–91)—have moved to the level of psychological primitives (26).

Although beyond the scope of the present manuscript, we will briefly outline this approach here to illustrate one potential way that we may understand self-injurious thoughts and behaviors on a level other than biopsychosocial factors. Psychological primitives are fundamental elements of the mind that cannot be reduced to anything else psychological (92). These psychological primitives give rise to all psychological phenomena. Three psychological primitives have been identified: internal stimuli, external stimuli, and conceptual knowledge (23, 91, 93, 94). Psychological phenomena (including behaviors) emerge when an individual attempts to make meaning of their current internal and external stimuli based on their conceptual knowledge (*i.e.*, prior experiences). For example, anger occurs when an individual makes sense of their ongoing internal and external stimuli based on their concept of anger. Each person's concept of anger is heterogenous (*i.e.*, includes many different exemplars of “anger”) and partially unique. As a result, there is substantial heterogeneity in the internal and external stimuli associated with anger, and in behavioral expressions of anger (95–97). This heterogeneity is why meta-analyses indicate that there is no neural or physiological signature for anger or any other emotion (98, 99). In other words, biopsychosocial factor associations with anger are complex.

The primitive-based approach makes sense of this complexity by proposing that this complex set of factors are all associated with anger *via* a common primitive-based mechanism: they all activate the anger concept. As a result, a major focus of the new primitive-based approach is to understand how concepts are formed, activated, implemented, and disrupted. For example, the anger concept can be disrupted with semantic satiation techniques, and this makes it more difficult for people to experience anger and to identify stereotypically angry faces (100, 101). Similarly, people with a certain form of semantic dementia do not possess concepts for specific emotions like anger. They are accordingly unable to distinguish between stereotypically angry, fearful, or sad faces (102). The primitive-based approach further specifies that all behaviors are motivated by allostasis (*i.e.*, prediction of whether the anticipated metabolic costs of a given behavior are worth the anticipated metabolic benefits; see 103). When an individual conceptualizes that a given behavior will promote allostasis more effectively than any other considered in a given moment, they engage in that behavior.

From this perspective, NSSI and suicide attempts are best understood in terms of concepts for NSSI and suicide, and momentary conceptualizations of how NSSI and/or suicide might contribute to allostasis. Based on this approach, self-injury concepts (*e.g.*, NSSI, suicide) are necessary (but not sufficient) for self-injury to occur. Consistent with this view, people who have immature self-injury concepts [*e.g.*, young children: (104, 105)] have very low rates of self-injurious thoughts and behaviors (4). As these concepts mature in late childhood and early adolescence (104, 105), the rates of self-injurious thoughts and behavior increase dramatically (4). Also based on this approach, the conceptualization that self-injury will promote allostasis more effectively than any other behavior in a given moment should be a necessary and sufficient cause of self-injurious behaviors. Recent work using a virtual reality (VR) suicide paradigm (106) is consistent with this possibility. These studies show that manipulations such as rejection, stress, and pain have little-to-no causal effect on VR suicide. But changing how someone conceptualizes the allostatic consequences of VR suicide (*e.g.*, if told that engaging in VR suicide will help one to avoid stress or pain, or to obtain a reward) has a large causal effect on VR suicide (106, 107). The greater the perceived likelihood of obtaining a reward or avoiding a punishment (*i.e.*, of promoting allostasis), the more likely someone is to engage in VR suicide (108).

From this perspective, self-injury theories should focus on how people develop self-injury concepts and how they arrive at the momentary conceptualization that self-injury will promote allostasis. Self-injury prediction efforts should focus on how people conceptualize the potential consequences of self-injury (*e.g.*, as providing major allostatic benefits *vs.* costs). And self-injury intervention efforts should focus on disrupting self-injury concepts and changing conceptualizations about the relative costs and benefits of engaging in self-injury. Once again, a full description of the primitive approach is beyond the scope of the present article (see 26 for a more detailed discussion), but the present findings along with the broader literature indicate that, clinically, we may benefit from developing primitive-based methods for predicting and preventing NSSI and suicidality.

A few limitations of the study should be considered when interpreting the findings. First, the present sample included individuals at high risk for self-injurious thoughts and behaviors. It is unclear how the findings might generate to other samples. Second, most participants in the NSSI group reported self-cutting as their primary form of NSSI. Future studies are needed to directly examine the differences between individuals primarily engaging in other forms of NSSI (*e.g.*, burning, scratching) and individuals engaging in suicide attempt. Because complexity already appeared to characterize the differences between the more uniform NSSI group (*i.e.*, primarily self-cutting) and the suicide attempt group, the current findings will likely replicate if the NSSI group is more heterogeneous. Third, the study was unable to include all factors associated with NSSI or suicide attempt. Although it is possible that future studies might discover one individual factor or a specific set of factors that is both necessary and sufficient to separate individuals engaging in suicide attempt from individuals who only engage in NSSI, it is increasingly implausible considering previous meta-analytic evidence (33, 34) and the present results.

In sum, the present study found that complex differences exist between individuals engaging in NSSI and those engaging in suicide attempt. It is always possible that future work will be able to constrain these differences to a complicated or simple set of factors. But we believe that it is most plausible to assume that these differences are truly complex and to shift some of our research questions and objectives to align with this complexity. One potential way to do this would be to move beyond biopsychosocial factors to a different level of analysis such as psychological primitives. Such a move may allow for biopsychosocial factor complexity while also providing an explanation for self-injurious thoughts and behaviors that is simple enough to advance theory, prediction, and treatment.

## Data Availability Statement

The datasets generated for this study are available on request to the corresponding author.

## Ethics Statement

The studies involving human participants were reviewed and approved by The Florida State University Human Subjects Office and Institutional Review Board. The patients/participants provided their written informed consent to participate in this study.

## Author Contributions

XH and JF conceived of the study. XH conducted analyses and drafted the initial manuscript. JR collected the data. JF and JR provided comments on the manuscript. XH and JF drafted the final manuscript.

## Funding

This study was supported in part by funding from the Military Suicide Research Consortium (MSRC), an effort supported by the Office of the Assistant Secretary of Defense for Health Affairs (Award No. W81XWH-10-2-0181). Opinions, interpretations, conclusions, and recommendations are those of the authors and are not necessarily endorsed by the funding agencies.

## Conflict of Interest

The authors declare that the research was conducted in the absence of any commercial or financial relationships that could be construed as a potential conflict of interest.

## References

[1] Nock MK Self-Injury. Annu Rev Clin Psychol (2010) 6:339–63. 10.1146/annurev.clinpsy.121208.131258 20192787

[2] Swannell SV Martin GE Page A Hasking P St John NJ Prevalence of nonsuicidal self-injury in nonclinical samples: Systematic review, meta-analysis and meta-regression. Suicide Life-Threat Behav (2014) 44:273–303. 10.1111/sltb.12070 24422986

[3] Baca-Garcia E Perez-Rodriguez M Keyes K Hasin D Suicidal ideation and suicide attempts in the United States. Mol Psychiatry (2011) 15:250–9. 10.1038/mp.2008.98.Suicidal PMC2825279 18779820

[4] Nock MK Green JG Hwang I McLaughlin KA Sampson NA Zaslavsky AM Prevalence, correlates, and treatment of lifetime suicidal behavior among adolescents: Results from the national comorbidity survey replication adolescent supplement. JAMA Psychiatry (2013) 70:300–10. 10.1001/2013.jamapsychiatry.55 PMC3886236 23303463

[5] Ribeiro JD Franklin JC Fox KR Bentley KH Kleiman EM Chang BP Self-injurious thoughts and behaviors as risk factors for future suicide ideation, attempts, and death: a meta-analysis of longitudinal studies. Psychol Med (2016). 46:225–336. 10.1017/S0033291715001804 26370729 PMC4774896

[6] Tarrier N Sommerfield C Pilgrim H Faragher B Factors associated with outcome of cognitive-behavioural treatment of chronic post-traumatic stress disorder. Behav Res Ther (2000) 38:191–202. 10.1016/S0005-7967(99)00030-3 10661003

[7] Berkman ND Lohr KN Bulik CM Outcomes of eating disorders: A systematic review of the literature. Int J Eat Disord (2007) 40:293–309. 10.1002/eat 17370291

[8] Joiner TE Why People Die by Suicide. Cambridge, MA: Harvard University Press (2005).

[9] Van Orden KA Witte TK Cukrowicz KC Braithwaite SR Selby EA Joiner TE The interpersonal theory of suicide. Psychol Rev (2010) 117:575–600. 10.1037/a0018697 20438238 PMC3130348

[10] Joiner TE Ribeiro JD Silva C Nonsuicidal Self-Injury, Suicidal Behavior, and Their Co-occurrence as Viewed Through the Lens of the Interpersonal Theory of Suicide. Curr Dir Psychol Sci (2012) 21:342–7. 10.1177/0963721412454873

[11] Ribeiro JD Franklin JC Fox KR Bentley KH Kleiman EM Chang BP Letter to the editor: suicide as a complex classification problem: machine learning and related techniques can advance suicide prediction - a reply to Roaldset (2016). Psychol Med (2016) 46:1–2. 10.1017/S0033291716000611 27091309

[12] Cilliers P Rules and complex systems. Crit Complex (2016) 2:40–50. 10.1515/9781501502590-006

[13] Mason PH Degeneracy at multiple levels of complexity. Biol Theory (2010) 5:277–88. 10.1162/biot_a_00041

[14] Pathak SD Day JM Nair A Sawaya WJ Kristal MM Complexity and adaptivity in supply networks: building supply network theory using a complex adaptive systems perspective. Decis Sci (2007) 38:547–80. 10.1111/j.1540-5915.2007.00170.x

[15] Poli R A Note on the Difference Between Complicated and Complex Social Systems. Cadmus (2013) 2:142–7.

[16] Whitacre J Bender A Degeneracy: a design principle for achieving robustness and evolvability. J Theor Biol (2010) 263:143–53. 10.1016/j.jtbi.2009.11.008 19925810

[17] Gelman SA Psychological essentialism in children. Trends Cognit Sci (2004) 8:404–9. 10.1016/j.tics.2004.07.001 15350241

[18] Read SJ Once is enough: Causal reasoning from a single instance. J Pers Soc Psychol (1983) 45:323–34. 10.1037/0022-3514.45.2.323

[19] Waldmann MR Combining versus analyzing multiple causes: how domain assumptions and task context affect integration rules. Cognit Sci (2007) 31:233–56. 10.1080/15326900701221231 21635296

[20] Edelman GM Gally JA Degeneracy and complexity in biological systems. Proc Natl Acad Sci U S A (2001) 98:13763–8. 10.1073/pnas.231499798 PMC61115 11698650

[21] Mason PH Domínguez DJF Winter B Grignolio A Hidden in plain view: Degeneracy in complex systems. BioSystems (2015) 128:1–8. 10.1016/j.biosystems.2014.12.003 25543071

[22] Miller JH Page SE Complex adaptive systems: An introduction to computational models of social life. Princeton, NJ: Princeton University Press (2009).

[23] Barrett LF The theory of constructed emotion : an active inference account of interoception and categorization. Soc Cognit Affect Neurosci (2017) 12:1–23. 10.1093/scan/nsw154 27798257 PMC5390700

[24] Barrett LF Satpute AB Historical pitfalls and new directions in the neuroscience of emotion. Neurosci Lett (2019) 693:9–18. 10.1016/j.neulet.2017.07.045 28756189 PMC5785564

[25] Cicchetti D Rogosch FA Equifinality and multifinality in developmental psychopathology. Dev Psychopathol (1996) 8:597–600. 10.1017/S0954579400007318

[26] Franklin JC Psychological primitives can make sense of biopsychosocial factor complexity in psychopathology. BMC Med (2019) 17:1–8. 10.1186/s12916-019-1435-1 31623620 PMC6798358

[27] Ribeiro JD Huang X Fox KR Walsh CG Linthicum KP Predicting imminent suicidal thoughts and nonfatal attempts: the role of complexity. Clin Psychol Sci (2019) 7:941–57. 10.1177/2167702619838464

[28] Klonsky ED Muehlenkamp JJ Self-Injury: A Research Review for the Practitioner. J Clin Psychol (2007) 63:1045–56. 10.1002/jclp 17932985

[29] Hauser DJ Schwarz N Attentive Turkers: MTurk participants perform better on online attention checks than do subject pool participants. Behav Res Methods (2016) 48:400–7. 10.3758/s13428-015-0578-z 25761395

[30] Casler K Bickel L Hackett E Separate but equal? A comparison of participants and data gathered via Amazon's MTurk, social media, and face-to-face behavioral testing. Comput Hum Behav (2013) 29:2156–60. 10.1016/j.chb.2013.05.009

[31] Hauser DJ Paolacci G Chadnler J Common concerns with MTurk as a participant pool: Evidence and solutions. In: Kardes FR Herr PP Schwarz N, editors. Handbook in Research Methods in Consumer Psychology. Abingdon, UK: Routledge (2019) p. 1–21.

[32] Shapiro DN Chandler J Mueller PA Using mechanical turk to study clinical populations. Clin Psychol Sci (2013) 1:213–20. 10.1177/2167702612469015

[33] Fox KR Franklin JC Ribeiro JD Kleiman EM Bentley KH Nock MK Meta-analysis of risk factors for nonsuicidal self-injury. Clin Psychol Rev (2015). 42:156–67 10.1016/j.cpr.2015.09.002 PMC4772426 26416295

[34] Franklin JC Ribeiro JD Fox KR Bentley KH Kleiman EM Huang X Risk factors for suicidal thoughts and behaviors : a meta-analysis of 50 years of research. Psychol Bull (2017) 143:187–232. 10.1037/bul0000084 27841450

[35] Nock MK Holmberg EB Photos VI Michel BD Self-Injurious Thoughts and Behaviors Interview: development, reliability, and validity in an adolescent sample. Psychol Assess (2007) 19:309–17. 10.1037/1040-3590.19.3.309 17845122

[36] Franklin JC Puzia ME Lee KM Prinstein MJ Low implicit and explicit aversion toward self-cutting stimuli longitudinally predict nonsuicidal self-injury. J Abnorm Psychol (2014) 123:463–9. 10.1037/a0036436 PMC4163747 24886018

[37] Franklin JC Fox KR Franklin CR Kleiman EM Ribeiro JD Jaroszewski AC A brief mobile app reduces nonsuicidal and suicidal self-injury: evidence from three randomized controlled trials. J Consult Clin Psychol (2016) 84:544–57. 10.1037/ccp0000093 27018530

[38] Ribeiro JD Witte TK Van Orden KA Selby EA Gordon KH Bender TW Fearlessness about death: the psychometric properties and construct validity of the revision to the Acquired Capability for Suicide Scale. Psychol Assess (2014) 26:115–126 12p. 10.1037/a0034858 24274043 PMC4093903

[39] Hendin H Al Jurdi RK Houck PR Hughes S Turner JB Role of intense affects in predicting short-term risk for suicidal behavior: a prospective study. J Nerv Ment Dis (2010) 198:220–5. 10.1097/NMD.0b013e3181d13d14 20216000

[40] Hendin H Maltsberger JT Szanto K The role of intense affective states in signaling a suicide crisis. J Nerv Ment Dis (2007) 195:363–8. 10.1097/NMD.0b013e318052264d 17502800

[41] Franklin JC Lee KM Puzia ME Prinstein MJ Recent and frequent nonsuicidal self-injury is associated with diminished implicit and explicit aversion toward self-cutting stimuli. Clin Psychol Sci (2014) 2:306–18. 10.1177/2167702613503140 PMC4163747 24886018

[42] Franklin J Puzia M Lee K Prinstein MJ Low implicit and explicit aversion toward self-cutting stimuli longitudinally predict nonsuicidal self-injury. J Abnorm Psychol (2014) 123:463–9. 10.1037/a0036436 PMC4163747 24886018

[43] Payne BK Cheng CM Govorun O Stewart BD An inkblot for attitudes: affect misattribution as implicit measurement. J Pers Soc Psychol (2005) 89:277–93. 10.1037/0022-3514.89.3.277 16248714

[44] Fox KR Ribeiro JD Kleiman EM Hooley JM Nock MK Franklin JC Affect toward the self and self-injury stimuli as potential risk factors for nonsuicidal self-injury. Psychiatry Res (2018) 260:279–85. 10.1016/j.psychres.2017.11.083 29223043

[45] Beck AT Kovacs M Weissman A Assessment of suicidal intention: The Scale for Suicide Ideation. J Consult Clin Psychol (1979) 47:343–52. 10.1037/0022-006X.47.2.343 469082

[46] Beck AT Steer RA Beck Scale for Suicide Ideation: Manual. San Antonio, TX: Psychological Corporation (1991).

[47] Ribeiro JD Bender TW Selby EA Hames JL Joiner TE Development and validation of a brief self-report measure of agitation: the Brief Agitation Measure. J Pers Assess (2011) 93:597–604. 10.1080/00223891.2011.608758 21999383

[48] Derogatis LR BSI 18, Brief Symptom Inventory 18: Administration, Scoring and Procedures Manual. Minneapolis, MN: NCS Pearson, Inc (2001).

[49] Lang PJ Bradley MM Cuthbert BN International Affective Picture System (IAPS): Affective Ratings of Pictures and Instruction Manual. Gainesville, FL: NIMH, Center for the Study of Emotion & Attention (2005).

[50] Fazio RH Jackson JR Dunton BC Williams CJ Variability in automatic activation as an unobtrusive measure of racial attitudes: a bona fide pipeline? J Pers Soc Psychol (1995) 69:1013–27. 10.1037/0022-3514.69.6.1013 8531054

[51] Nier JA How dissociated are implicit and explicit racial attitudes? A Bogus Pipeline approach. Gr Process Intergr Relations (2005) 8:39–52. 10.1177/1368430205048615

[52] Ribeiro JD Bodell L Joiner TE in Poster Presented at the 46th Annual Meeting of the Association for Behavioral and Cognitive Therapies, National Harbor, MD, (2012), November.

[53] Chu C Bodell LP Ribeiro JD Joiner TE Eating disorder symptoms andsuicidal ideation: The moderating role of disgust. Eur Eat Disord Rev (2015) 23:545–52. 10.1002/erv.2373 26010299

[54] Bastien CH Vallieres A Morin CM Validation of the Insomnia Severity Index as an outcome measure for insomnia research. Sleep Med (2001) 2:297–307. 10.1080/15402002.2011.606766 11438246

[55] Bernert RA Joiner TE Cukrowicz KC Schmidt NB Krakow B Suicidality and Sleep Disturbances. Sleep (2005) 28:1135–41. 10.1093/sleep/28.9.1135 16268383

[56] Andover MS Gibb BE Non-suicidal self-injury, attempted suicide, and suicidal intent among psychiatric inpatients. Psychiatry Res (2010) 178:101–5. 10.1016/j.psychres.2010.03.019 20444506

[57] Jacobson CM Muehlenkamp JJ Miller AL Turner JB Psychiatric impairment among adolescents engaging in different types of deliberate self-harm. J Clin Child Adolesc Psychol (2008) 37:363–75. 10.1080/15374410801955771 18470773

[58] R Core Team R: A language and environment for statistical computing. Vienna, Austria: R Foundation for Statistical Computing (2019).

[59] Nock MK Millner AJ Joiner TE Gutierrez PM Han G Hwang I Risk factors for the transition from suicide ideation to suicide attempt: results from the army study to assess risk and resilience in Servicemembers (Army STARRS). J Abnorm Psychol (2018) 127:139–49. 10.1037/abn0000317 PMC5851467 29528668

[60] Zuromski KL Bernecker SL Gutierrez PM Joiner TE King AJ Liu H Assessment of a risk index for suicide attempts among US army soldiers with suicide ideation: analysis of data from the Army Study to Assess Risk and Resilience in Servicemembers (Army STARRS). JAMA Netw Open (2019) 2:e190766. 10.1001/jamanetworkopen.2019.0766 30874786 PMC6484656

[61] An S Malhotra K Dilley C Han-Burgess E Valdez JN Robertson J Predicting drug-resistant epilepsy — A machine learning approach based on administrative claims data. Epilepsy Behav (2018) 89:118–25. 10.1016/j.yebeh.2018.10.013 PMC6461470 30412924

[62] Fox KR Huang X Linthicum KP Wang SB Franklin JC Ribeiro JD Model complexity improves the prediction of nonsuicidal self-injury. J Consult Clin Psychol (2019) 87:684–92. 10.1037/ccp0000421.supp 31219275

[63] Singal AG Mukherjee A Joseph Elmunzer B Higgins PDR Lok AS Zhu J Machine learning algorithms outperform conventional regression models in predicting development of hepatocellular carcinoma. Am J Gastroenterol (2013) 108:1723–30. 10.1038/ajg.2013.332 PMC4610387 24169273

[64] Walsh CG Ribeiro JD Franklin JC Predicting risk of suicide attempts over time through machine learning. Clin Psychol Sci (2017) 5:457–69. 10.1177/2167702617691560

[65] James G Witten D Hastie T Tibshirani R An Introduction to Statistical Learning. New York, NY: Springer (2013).

[66] Breiman L Random forests. Mach Learn (2001) 45:5–32. 10.1023/A:1010933404324

[67] Breiman L Manual On Setting Up, Using, And Understanding Random Forests V3.1. Berkeley, CA: Department of Statistics, University of California, Berkeley (2002). Available at: https://www.stat.berkeley.edu/~breiman/Using_random_forests_V3.1.pdf.

[68] Meisner A Kerr KF Thiessen-Philbrook H Coca SG Parikh CR Methodological issues in current practice may lead to bias in the development of biomarker combinations for predicting acute kidney injury. Kidney Int (2016) 89:429–38. 10.1038/ki.2015.283 PMC4805513 26398494

[69] Moons KGM de Groot JAH Bouwmeester W Vergouwe Y Mallett S Altman DG Critical appraisal and data extraction for systematic reviews of prediction modelling studies: The CHARMS Checklist. PLoS Med (2014) 11:1–17. 10.1371/journal.pmed.1001744 PMC4196729 25314315

[70] Moons KGM Kengne AP Woodward M Royston P Vergouwe Y Altman DG Risk prediction models: I. Development, internal validation, and assessing the incremental value of a new (bio)marker. Heart (2012) 98:683–90. 10.1136/heartjnl-2011-301246 22397945

[71] Steyerberg EW Vergouwe Y Towards better clinical prediction models: Seven steps for development and an ABCD for validation. Eur Heart J (2014) 35:1925–31. 10.1093/eurheartj/ehu207 PMC4155437 24898551

[72] Jacobucci R Littlefield AK Millner AJ Kleiman EM Steinley D Pairing machine learning and clinical psychology: how you evaluate predictive performance matters. PsyArXiv (2020). 1–36 10.31234/osf.io/2yber

[73] Steyerberg EW Clinical Prediction Models. Cham, Switzerland: Springer International Publishing (2019).

[74] Steyerberg EW Bleeker SE Moll HA Grobbee DE Moons KGM Internal and external validation of predictive models: a simulation study of bias and precision in small samples. J Clin Epidemiol (2003) 56:441–7. 10.1016/S0895-4356(03)00047-7 12812818

[75] Maros ME Capper D Jones DTW Hovestadt V von Deimling A Pfister SM Machine learning workflows to estimate class probabilities for precision cancer diagnostics on DNA methylation microarray data. Nat Protoc (2020) 15:479–512. 10.1038/s41596-019-0251-6 31932775

[76] Zhang Y Wang Y Zhou W Fan Y Zhao J Zhu L A combined drug discovery strategy based on machine learning and molecular docking. Chem Biol Drug Des (2019) 93:685–99. 10.1111/cbdd.13494 30688405

[77] Austin PC Lee DS Steyerberg EW Tu JV Regression trees for predicting mortality in patients with cardiovascular disease: what improvement is achieved by using ensemble-based methods? Biometrical J (2012) 54:657–73. 10.1002/bimj.201100251 PMC3470596 22777999

[78] Van Der Ploeg T Datema F Baatenburg De Jong R Steyerberg EW Prediction of survival with alternative modeling techniques using pseudo values. PLoS One (2014) 9:1–10. 10.1371/journal.pone.0100234 PMC4065009 24950066

[79] McKernan LC Lenert MC Crofford LJ Walsh CG Outpatient engagement and predicted risk of suicide attempts in fibromyalgia. Arthritis Care Res (2019) 71:1255–63. 10.1002/acr.23748 PMC6405325 30192068

[80] Ruderfer DM Walsh CG Aguirre MW Tanigawa Y Ribeiro JD Franklin JC Significant shared heritability underlies suicide attempt and clinically predicted probability of attempting suicide. Mol Psychiatry (2019), 1–9. 10.1038/s41380-018-0326-8 PMC6609505 30610202

[81] Walsh CG Ribeiro JD Franklin JC Predicting suicide attempts in adolescents with longitudinal clinical data and machine learning. J Child Psychol Psychiatry (2018) 59:1261–70. 10.1111/jcpp.12916 29709069

[82] Lindhiem O Petersen IT Mentch LK Youngstrom EA The Importance of Calibration in Clinical Psychology. Assessment (2018) 1–15. 10.1177/1073191117752055 PMC6778000 29457474

[83] Brier GW Verification of forecasts expressed in terms of probabilities. Mon Weather Rev (1950), 78: 1–3. 10.1175/1520-0493(1950)078<0001:VOFEIT>2.0.CO;2

[84] Barak-Corren Y Castro VM Javitt S Hoffnagle AG Dai Y Perlis RH Predicting suicidal behavior from longitudinal electronic health records. Am J Psychiatry (2017) 174:154–62. 10.1176/appi.ajp.2016.16010077 27609239

[85] Just MA Pan L Cherkassky VL McMakin DL Cha C Nock MK Machine learning of neural representations of suicide and emotion concepts identifies suicidal youth. Nat Hum Behav (2017) 1:911–9. 10.1038/s41562-017-0234-y PMC5777614 29367952

[86] Kessler RC Warner CH Ivany C Petukhova MV Rose S Bromet EJ Predicting suicides after psychiatric hospitalization in US army soldiers: The Army Study to Assess Risk and Resilience in Servicemembers (Army STARRS). JAMA Psychiatry (2015) 72:49–57. 10.1001/jamapsychiatry.2014.1754 25390793 PMC4286426

[87] Simon GE Stewart C Yarborough BJ Lynch F Coleman KJ Beck A Mortality rates after the first diagnosis of psychotic disorder in adolescents and young adults. JAMA Psychiatry (2018) 75:254–60. 10.1001/jamapsychiatry.2017.4437 PMC5885951 29387876

[88] Fox KR Huang X Guzman EM Funsch KM Cha CB Ribeiro JD How good are interventions of self-injurious thoughts and behaviors? A meta-analysis of 345 randomized controlled trials. In: . The Annual Meeting of the Association for Behavioral and Cognitive Therapies. Atlanta, GA The Association for Behavioral and Cognitive Therapies (2019) Available at: https://eventscribe.com/2019/ABCT/fsPopup.asp?Mode=presInfo&PresentationID=603431.

[89] Barrett LF The future of psychology: Connecting mind to brain. Perspect Psychol Sci (2009) 4:326–39. 10.1111/j.1745-6924.2009.01134.x PMC2763392 19844601

[90] Barrett LF Emotions Are Real. Emotion (2012) 12:413–29. 10.1037/a0027555 22642358

[91] Wilson-Mendenhall CD Barrett LF Barsalou LW Grounding Emotion in Situated Conceptualization. Neuropsychologia (2012) 49:1105–27. 10.1016/j.neuropsychologia.2010.12.032.Grounding PMC3078178 21192959

[92] Barrett LF Variety is the spice of life: A psychological construction approach to understanding variability in emotion. Cognit Emot (2009) 23:1284–306. 10.1080/02699930902985894 PMC2835153 20221411

[93] Barrett LF Bliss-Moreau E Affect as a psychological primitive. Adv Exp Soc Psychol (2009) 41:167–218. 10.1016/S0065-2601(08)00404-8 20552040 PMC2884406

[94] Barrett LF Solving the emotion paradox: Categorization and the experience of emotion. Pers Soc Psychol Rev (2006) 10:20–46. 10.1207/s15327957pspr1001_2 16430327

[95] Barrett LF Mesquita B Ochsner KN Gross JJ The Experience of Emotion. Annu Rev Psychol (2007) 58:373–403. 10.1146/annurev.psych.58.110405.085709 17002554 PMC1934613

[96] Wormwood JB Neumann AE Barrett LF Quigley KS Understanding emotion in context: how the Boston marathon bombings altered the impact of anger on threat perception. J Appl Soc Psychol (2017) 47:13–22. 10.1111/jasp.12412

[97] Boiger M Mesquita B Uchida Y Barrett LF Condoned or condemned: The situational affordance of anger and shame in the United States and Japan. Pers Soc Psychol Bull (2013) 39:540–53. 10.1177/0146167213478201 23471319

[98] Siegel EH Sands MK Van den Noortgate W Condon P Chang Y Dy J Emotion fingerprints or emotion populations? A meta-analytic investigation of autonomic features of emotion categories. Psychol Bull (2018) 144:343–93. 10.1037/bul0000128 PMC5876074 29389177

[99] Lindquist KA Wager TD Kober H Bliss-Moreau E Barrett LF The brain basis of emotion: A meta-analytic review. Behav Brain Sci (2012) 35:121–43. 10.1017/S0140525X11000446 PMC4329228 22617651

[100] Lindquist KA Barrett LF Bliss-Moreau E Russell JA Language and the perception of emotion. Emotion (2006) 6:125–38. 10.1037/1528-3542.6.1.125 16637756

[101] Gendron M Lindquist KA Barsalou L Barrett LF Emotion words shape emotion percepts. Emotion (2012) 12:314–25. 10.1037/a0026007 PMC4445832 22309717

[102] Lindquist KA Gendron M Barrett LF Dickerson BC Emotion perception, but not affect perception, is impaired with semantic memory loss. Emotion (2014) 14:375–87. 10.1037/a0035293 PMC4119962 24512242

[103] Touroutoglou A Andreano JM Adebayo M Lyons S Barrett LF Motivation in the Service of Allostasis: The Role of Anterior Mid-Cingulate Cortex. In: Elliot A, editor. Advances in Motivation Science. p. 1–25. 10.1016/bs.adms.2018.09.002 PMC6884085 31788441

[104] Cuddy-Casey M Orvaschel H Children's understanding of death in relation to child suicidality and homicidality. Clin Psychol Rev (1997) 17:33–45. 10.1016/S0272-7358(96)00044-X 9125366

[105] Mishara BL Conceptions of death and suicide in children ages 6-12 and their implications for suicide prevention. Suicide Life-Threat Behav (1999) 29:105–18. 10.1111/j.1943-278X.1999.tb01049.x 10407964

[106] Franklin JC Huang X Bastidas D Development of a translational approach for studying suicide causes. Behav Res Ther (2019) 120:1–10. 10.1016/j.brat.2018.12.013 30616833

[107] Huang X Franklin JC Virtual reality suicide: A new method for identifying suicidality causes and treatment targets. In: International Summit of Suicide Resarch. Miami, FL (2019). Available at: https://pmg.joynadmin.org/documents/1013/5daf102c68ed3f216d2a47f6.pdf.

[108] Linthicum KP Harris L Ribeiro JD But, what if?: An experimental study of the effects of uncertainty on suicidal behavior. In: International Summit of Suicide Resarch. Miami, FL (2019). Available at: https://pmg.joynadmin.org/documents/1013/5daf102c68ed3f216d2a47f6.pdf.

